# Appearance of Ankylosing Spondylitis in a Middle-Aged Female Patient With a Long History of Rheumatoid Arthritis

**DOI:** 10.7759/cureus.34952

**Published:** 2023-02-13

**Authors:** Ryuichi Ohta, Chiaki Sano

**Affiliations:** 1 Community Care, Unnan City Hospital, Unnan, JPN; 2 Community Medicine Management, Shimane University Faculty of Medicine, Izumo, JPN

**Keywords:** ankylosing spondylitis, general medicine, rural hospital, female, axial spondyloarthritis, rheumatoid arthritis

## Abstract

Ankylosing spondylitis (AS) is a chronic inflammatory disease of the axial bones, primarily affecting the spine. AS is classified as spondyloarthritis because it affects the spine and other joints. AS has several presentations and, in some cases, can be insidious, making it difficult to diagnose. We encountered a patient on long-term follow-up for rheumatoid arthritis with an appearance of AS. This case suggests that patients with long-term rheumatoid arthritis can develop AS during follow-up and that the seropositivity of rheumatoid factors and anti-cyclic citrullinated peptide antibodies cannot rule out AS. Therefore, the possibility of AS should be considered, even in patients diagnosed with rheumatoid arthritis.

## Introduction

Ankylosing spondylitis (AS) is a chronic inflammatory disease of the axial bones, primarily affecting the spine. AS is classified as spondyloarthritis because it affects the spine and other joints [1]. Symptoms of AS include back pain and stiffness, especially in the morning or after sitting or lying down for prolonged periods [1]. These symptoms cause inflammatory back pain, resulting in inflammatory diseases. Inflammatory back pain occurs in 70-80% of patients with AS [1]. In addition, the disease can cause deformities and fusion of the spinal vertebrae, leading to loss of mobility and requiring prompt diagnosis and treatments [2].

AS has several presentations and, in some cases, can be insidious, making it difficult to diagnose. The Assessment of Spondyloarthritis International Society criteria can be used to evaluate patient symptoms, physical examination results, and imaging results to make a diagnosis [3]. Physical examination reveals stiffness and inflammation in the spine and other joints [2]. However, X-rays can detect characteristic changes in the spine, such as bone spurs and fusion of the vertebrae [4]. Blood tests can detect systemic inflammation with C-reactive protein, erythrocyte sedimentation rate, and human leukocyte antigen (HLA)-B27 gene abnormalities. However, HLA-B27 levels cannot be measured in the Japanese health insurance system. Magnetic resonance imaging (MRI) and computed tomography can reveal inflammation and damage to the spine and other joints [1].

The coexistence of other collagenous diseases can lead to a delayed diagnosis of AS. Here, we report a case of rheumatoid arthritis followed by AS. Previous studies have shown that AS is followed by rheumatoid arthritis [5]. However, to date, only a few cases of rheumatoid arthritis followed by AS have been reported. We encountered a patient on a long-term follow-up for rheumatoid arthritis with the appearance of AS. In this case, we discuss that early closure of differential diagnosis should be done with caution in rheumatology, and anchoring bias should be avoided for early mitigation of patient symptoms [6].

## Case presentation

A 52-year-old female visited the outpatient department of a rural community hospital with the chief complaint of new-onset back and hand pain for six weeks. She noticed back and hand pain six weeks ago when waking up in the morning. Motion exacerbated the pain after resting; however, the pain improved with continuous movement. She did not experience night sweats, fever, weight loss, or eye or finger pain. She had a medical history of osteoarthritis in her hand and rheumatoid arthritis for 10 years with methotrexate (MTX) (14 mg per week) and tacrolimus (1.5 mg per day) treatments.

Vital signs at the visit were as follows: blood pressure of 110/75 mmHg; pulse rate of 65 beats/minute; body temperature of 36.7°C; respiratory rate of 16 breaths/minute; and oxygen saturation of 96% on room air. The patient was oriented toward time, place, and person. Physical examination revealed tenderness in both sacroiliac joints. Extremities examinations did not reveal any swelling, erythema, or tenderness of either hand's proximal interphalangeal (PIP) joints. No other neurological abnormalities were observed. There were no apparent abnormalities in the chest or abdomen and no skin eruptions. Laboratory tests showed mild inflammation exacerbation and no liver or kidney abnormalities (Table 1).

**Table 1:** Initial laboratory data of the patient eGFR, estimated glomerular filtration rate; CK, creatine kinase; CRP, C-reactive protein; Ig, immunoglobulin; HCV, hepatitis C virus; SARS-CoV-2, severe acute respiratory syndrome coronavirus 2; HBs, hepatitis B surface antigen; HBc, hepatitis B core antigen; C3, complement component 3; C4, complement component 4; MPO-ANCA, myeloperoxidase-antineutrophil cytoplasmic antibody; CCP, cyclic citrullinated peptide.

Parameters	Level	Reference
White blood cells	6.0	3.5–9.1 × 10^3^/μL
Neutrophils	64.1	44.0–72.0%
Lymphocytes	28.1	18.0–59.0%
Monocytes	5.5	0.0–12.0%
Eosinophils	1.3	0.0–10.0%
Basophils	1.0	0.0–3.0%
Red blood cells	3.90	3.76–5.50 × 10^6^/μL
Hemoglobin	12.7	11.3–15.2 g/dL
Hematocrit	38.7	33.4–44.9%
Mean corpuscular volume	98.8	79.0–100.0 fL
Platelets	31.4	13.0–36.9 × 10^4^/μL
Total protein	6.9	6.5–8.3 g/dL
Albumin	4.4	3.8–5.3 g/dL
Total bilirubin	0.3	0.2–1.2 mg/dL
Aspartate aminotransferase	28	8–38 IU/L
Alanine aminotransferase	27	4–43 IU/L
Alkaline phosphatase	83	106–322 U/L
Lactate dehydrogenase	185	121–245 U/L
Blood urea nitrogen	16.8	8–20 mg/dL
Creatinine	0.54	0.40–1.10 mg/dL
eGFR	90	>60.0 mL/min/L
Serum Na	141	135–150 mEq/L
Serum K	4.1	3.5–5.3 mEq/L
Serum Cl	107	98–110 mEq/L
CK	22	56–244 U/L
CRP	0.33	<0.30 mg/dL
IgG	966	870–1700 mg/dL
IgM	89	35–220 mg/dL
IgA	155	110–410 mg/dL
IgE	21	<173 mg/dL
HBs antigen	0.0	IU/mL
HBs antibody	0.00	mIU/mL
HBc antibody	0.00	S/CO
SARS-CoV-2 antigen	Negative	Negative
C3	125	86–164 mg/dL
C4	29	17–45 mg/dL
MPO-ANCA	<1.0	<3.5 U/mL
Rheumatoid factor	120	<5 IU/mL
Anti-CCP antibody	5.4	<5 U/mL
Anti-cardiolipin antibody IgG	<4	<4 U/mL
Urine test		
Leukocyte	Negative	Negative
Nitrite	Negative	Negative
Protein	Negative	Negative
Glucose	Negative	Negative
Urobilinogen	Normal	
Bilirubin	Negative	Negative
Ketone	Negative	Negative
Blood	Negative	Negative
pH	7.0	
Specific gravity	1.010	

**Figure 1:**
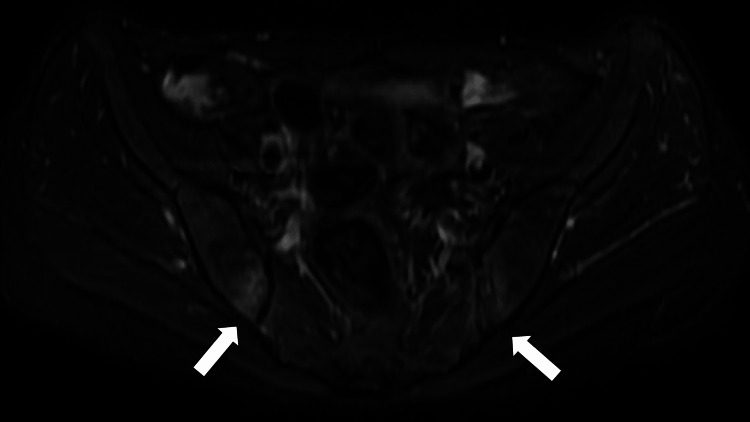
Pelvic magnetic resonance imaging (short inversion time inversion recovery) showing a high-intensity lesion on the joints, indicating the presence of sacroiliac joints inflammation (white arrows)

Initially, we considered that she might have had an exacerbation of osteoarthritis or rheumatoid arthritis because she visited the outpatient department frequently due to joint pain; therefore, we prescribed diclofenac as a pain reliever for two weeks. Her pain was partially alleviated but persisted. Two weeks later, pelvic radiography revealed bilateral sacroiliac joint deformation. Other pelvic MRI (short inversion time inversion recovery) showed a high-intensity lesion on the joints, indicating the presence of sacroiliac joint inflammation (Figure 1).

A follow-up physical examination revealed limited flexion and extension of the spinal joints. Finally, we diagnosed her with AS and started subcutaneous etanercept at 50 mg/week, which improved her pain and quality of life.

## Discussion

This case suggests that patients with long-term rheumatoid arthritis can develop AS during follow-up and that the seropositivity of rheumatoid factors and anti-cyclic citrullinated peptide (anti-CCP) antibodies cannot rule out AS. Therefore, the diagnosis of rheumatological diseases should include various differential diagnoses, even in patients already diagnosed with rheumatological diseases [6].

The coexistence of rheumatoid arthritis and AS is not common, but it is possible based on the pathophysiology, including cytokine interactions in each disease. Rheumatoid arthritis can be triggered mainly by aberrant lymphocytes with increased levels of cytokines, such as tumor necrosis factor (TNF) and interleukins (IL) 1 and 6 [7]. These cytokines can be controlled by disease-modifying antirheumatic drugs (DMARDs) [8]. Pathophysiologically, AS can be triggered by innate immune cells such as macrophages and neutrophils, along with cytokines such as TNF and IL-1, 12, 17, and 23 [1]. In addition, TNFs are common in these two diseases. Although there are differences in aberrant immune cells, increasing TNF can stimulate various immune cells systematically, resulting in both diseases [9]. In our case, the patient had chronic rheumatoid arthritis. Therefore, chronic inflammation may have triggered inflammation of the sacroiliac joints, leading to the diagnosis of AS.

A diagnosis of AS may require constant follow-up, even in patients with rheumatoid arthritis. In this case, the patient had new-onset back and joint pain with morning stiffness during long-term treatment of rheumatoid arthritis with MTX. Methotrexate can treat peripheral arthritis in AS but is ineffective in enthesitis [1]. Even in this case, the appearance of AS during chronic rheumatoid arthritis treatment may indicate MTX's ineffectiveness in treating AS. Additionally, PIP joint pain is common among middle-aged and older patients because of the high prevalence of osteoarthritis [10]. Here, we initially misdiagnosed the patient with osteoarthritis. Previous reports suggest that ultrasound can differentiate enthesitis from osteoarthritis, but subtle inflammation may not be detected, depending on the experience of the operators and the function of the ultrasound [11].

Especially in rural contexts with a lack of resources, rheumatologists need to consider the appearance of AS in patients with rheumatoid arthritis who are being treated with conventional synthetic DMARDs and new-onset axial pain and stiffness during long-term follow-up. In addition, in rural contexts, the rate of rheumatic diseases in older patients is increasing, and these patients have vague symptoms [12]. In such cases, differentiating between osteoarthritis, AS, and rheumatoid arthritis can be challenging. Furthermore, among older patients, the differentiation between seronegative rheumatoid arthritis and polymyalgia rheumatica is challenging because of the similarity of symptoms between them [13]. Therefore, rural rheumatologists should examine each patient through detailed history taking and physical examinations to avoid diagnostic biases [3].

## Conclusions

Patients with long-term rheumatoid arthritis can develop AS during a long follow-up period, and seropositivity for rheumatoid factors and anti-CCP antibodies cannot rule out AS. Therefore, the diagnosis of rheumatic diseases should include various differential diagnoses, even in patients already diagnosed with rheumatic diseases. In rural contexts, older patients with rheumatological diseases show vague symptoms; therefore, rural rheumatologists should comprehensively investigate these symptoms during follow-up.
